# Body composition and cancer survival: a narrative review

**DOI:** 10.1038/s41416-023-02470-0

**Published:** 2023-10-27

**Authors:** Patrick T. Bradshaw

**Affiliations:** School of Public Health, Division of Epidemiology, University of California Berkeley, Berkeley, CA USA

**Keywords:** Cancer epidemiology, Epidemiology

## Abstract

Interest in understanding the relationship between body composition and cancer survival has remained strong for decades, with a number of recent systematic reviews on the topic. However, the current state of evidence is based on heterogeneous exposure definitions based on anthropometry, yielding inconsistent findings with regard to this association. Recently the field has taken an exciting direction with the application of radiological assessments to measure specific aspects of body composition, yet reconciliation of findings from these modern assessment tools with those from the historic use of anthropometric data proves challenging. In this paper, I briefly review the biological basis for a link between body composition and cancer survival and summarize the epidemiological evidence with consideration to specific exposure measures. As enthusiasm is building around novel assessments, I conclude with a discussion of issues that researchers should be aware of when interpreting results from these new modalities.

## Introduction

The relationship between body composition and cancer survival has been investigated for decades, as noted by a recent review and meta-analysis that included results published over 30 years ago [1]. Most previous studies in this area have focused on the characteristic of excess adiposity, typically assessed with metrics derived from anthropometric measurements [1–3]. The specific interest in the association between survival outcomes and fat mass stems from the understanding that excess adiposity is a risk factor for a number of high-burden cancers [4], the plausible biological mechanisms that may link it to cancer survival [5–8], and its relationships with other high-burden comorbidities experienced by cancer patients such as diabetes and cardiovascular disease [9, 10]. These connections are especially concerning given the rapid increase in obesity prevalence among cancer survivors [11], but also because some cancer survivors experience significant weight gain around and immediately after diagnosis [12, 13]. Despite their prolific use, anthropometric measures of body composition have well-known limitations [14] which may play a role in apparently paradoxical findings noted in the body composition literature [15]. In response, researchers have recently shifted focus to more direct measures of both fat and muscle tissue from clinical assessments that are able to capture the amount and characteristics of the quality of various tissues simultaneously. While there are plausible links suggesting that elevated adiposity is linked to greater risk of death, there is similarly strong evidence suggesting greater lean mass, in particular muscle, is associated with a reduction in risk [16]. A better understanding of the multi-dimensional nature of the body composition-survival relationship would help resolve some of the ongoing confusion in the field [15, 17].

In this report, I will begin by reviewing the relevant biological mechanisms thought to link adiposity and muscle tissue to cancer survival. I will then summarize the epidemiological evidence of the relationship between survival and several common measures of body composition across cancer sites. Throughout the paper I will highlight important considerations for these different body composition metrics regarding the assessment and interpretation of the associations.

## Potential mechanisms

**Fig. 1:**
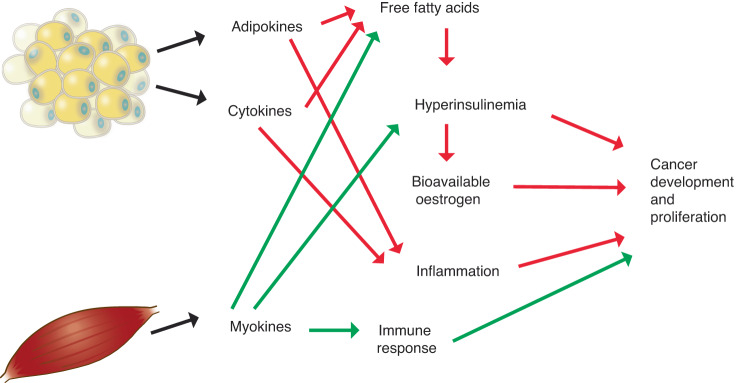
Putative mechanisms linking body composition features to cancer outcomes. Green arrows denote beneficial direction of associations, red arrows denote deleterious direction of associations.

As illustrated in Fig. 1, adipose tissue, especially visceral fat, is metabolically active and has a number of sequelae that are believed to influence the etiology and prognosis of several cancers in a complex interplay [18]. Excess adiposity is associated with higher levels of several mitogenic factors, such as insulin and insulin like growth factors [19, 20] which can encourage proliferation of cancerous cells. An increase in fat mass is associated with elevated levels of serum free fatty acids through several mechanisms that encourage lipolysis. One such mechanism is that visceral adipose tissue is less sensitive to the antilipolytic effect of insulin and more sensitive to the lipolytic effects of catecholamines [21]; this may be particularly important for cancer patients as catecholamine levels are increased by psychosocial stress, surgery, and treatment [22]. Adipocytes are also known to secrete a variety of cytokines including the lipolysis stimulating tumor necrosis factor alpha (TNF-α) [23]. The increase in free fatty acids driven by adipocytes in both subcutaneous and visceral adipose tissue is thought to inhibit insulin’s effect on glucose uptake and oxidation [24] thereby resulting in a state of insulin resistance, and a subsequent compensatory increase in insulin secretion by the pancreas in an effort to maintain glucose homeostasis [25]. This increase in insulin precipitates a decrease in insulin-like growth factor binding proteins (IGF-BPs) and a successive increase in bioavailable insulin-like growth factor I (IGF-I) [25]. Both insulin and IGF-I, as well as TNF-α, bind to membrane-bound receptors on cells that stimulate cellular proliferation and inhibit apoptosis, thereby providing a mechanism for tumor development [18, 26]. This pathway is especially relevant to certain cancers, such as breast [27], as mammary cell carcinomas typically exhibit an over-expression of insulin receptors [28] and IGF-I receptors [29] making them very susceptible to the proliferative effects of these hormones. Although visceral fat has been implicated in obesity-related insulin resistance, there is evidence that subcutaneous fat, specifically deep subcutaneous adipose tissue, may also play a role. Subcutaneous fat around the abdomen is comprised of two layers of superficial and deep tissue separated by fascia [30]. Deep subcutaneous adipose tissue shares several similarities with visceral adipose tissue including a comparable fatty acid composition [31] and a strong association with insulin resistance [32]. This suggests the mechanisms mentioned above may also be relevant for certain patterns of subcutaneous fat distribution.

Obesity-related inflammation may also provide a pathway through which excess adiposity influences carcinogenesis [33, 34]. In addition to TNF-α, inflammatory markers produced in response to excess adipose tissue include C-reactive protein (CRP) and interleukin-6 (IL-6), all of which have been implicated in carcinogenesis through various mechanisms [18]. Obesity is also involved in the regulation of other adipokines with the potential for tumor promotion [35]. Circulating levels of adiponectin, an adipocyte-specific protein with anti-inflammatory and insulin sensitizing effects [36], are lower in obese individuals while levels of leptin, which has potential to act as a growth factor, are positively related to adiposity [35]. In addition to the systemic inflammatory effects of elevated adiposity, mounting evidence suggests that fat cells surrounding the tumor may have important influences on local inflammation in the tumor microenvironment [18, 34]. This local inflammation is especially relevant for malignancies that occur in close proximity to adipose tissue depots, such as breast cancer [34].

Sex hormones are powerful mitogens which stimulate cellular proliferation therefore increasing the likelihood of a DNA mutation during cell division and encouraging replication of aberrant cells [37–39]. Aromatization of androgens in adipose tissue yields estrone which is subsequently converted to estradiol, the most metabolically active estrogen [40]. This pathway represents a significant source of estrogen for males and postmenopausal females, in contrast to premenopausal women where ovarian production of estradiol overshadows adipose-mediated formation [21]. The association between fat mass and sex hormones and related binding proteins, especially estradiol and sex hormone binding globulin (SHBG), are thought to play a significant role in carcinogenesis [41]. The availability of estradiol to target tissues is primarily determined by the amount of circulating SHBG. Approximately half of the estradiol in the blood is bound to SHBG, the remainder bound to albumin or freely circulating [42] A common consequence of obesity-related hyperinsulinemia is a reduction in SHBG, resulting in an increase in bioavailable estrogen allowing more free or albumin-bound estradiol to bind with estrogen receptors [41]. The combined effect of unregulated estradiol exposure and reduction in SHBG has been shown to result in a greater than two-fold increase in free estradiol among obese postmenopausal women compared to women of normal weight [42]. Excess adiposity, assessed by body mass index (BMI, weight in kilograms divided by squared height in meters), has been shown to be positively associated with estrone, estradiol, free estradiol, free testosterone and prolactin and negatively associated with SHBG [21, 43]. Besides its mitogenic potential, there is also evidence that estrogen metabolism generates free radicals which may inflict DNA damage thereby initiating carcinogenesis [44, 45]. The influence of obesity-driven hormone dysregulation is particularly relevant for treatment of cancers with a hormonal etiology. In particular, aromatase inhibitor therapy has been shown to be less effective in female breast cancer patients with obesity [46]. In addition, while adiposity is associated with lower testosterone levels in males [47], increased exposure to obesity-related growth factors and adipokines is related to activation of androgen receptors which may influence prostate cancer progression [48].

In contrast to adipose tissue, muscle mass is associated with a generally favorable metabolic and inflammatory profile. Muscle cells produce a number of proteins called myokines that have anti-inflammatory and insulin-sensitizing influences in opposition to the effects of adipokines [49]. These, and other factors, may be due in part to the relationship between muscle and physical activity [50]. Physical activity is associated with an increase in insulin sensitivity by increasing expression of the GLUT-4 glucose transporter in the plasma membrane of skeletal muscle [51–55] and by reducing the level of free fatty acids, which have been linked to impaired insulin function [56]. This increase in insulin sensitivity precipitates a decrease in insulin secretion, which is a possible mechanism for the observed increase in IGF-BPs [57] and decrease in IGF observed among physically active individuals [58]. The ability of physical activity to mediate these metabolic hormones and growth factors suggests another potential pathway for the observed protective effect of this exposure [59, 60]. This reduction in IGF may yield additional cancer protection as it may reduce sex hormone exposure by encouraging an increase in SHBG production by the liver [61]. The beneficial effects of physical activity on cancer outcomes may also include improvement of the immune response [62]. Regular physical activity has been associated with increases in number and cytotoxicity of natural killer cells, as well as favorable shifts in several inflammatory markers including IL-6, CRP, and TNF-α [63].

Obscuring our understanding of the muscle and cancer survival relationship is the fact that muscle mass is often altered by the presence of malignancy. Tumor-driven inflammation precipitates a catabolic condition known as cachexia [64], which results in loss of both fat and muscle tissue [16]. Muscle loss often culminates in a state referred to as sarcopenia, which should be noted can also manifest in the absence of cachexia and is frequently observed in aging populations [65]. Importantly, cancer patients often present with a high proportion of adipose tissue and low amount of muscle mass together, a condition termed “sarcopenic obesity” [16, 66]. This combination of low muscle and high fat is associated with greater systemic inflammation and metabolic dysfunction [67–69], which complicates the interpretation of the relationship between survival and the individual tissue measures. Importantly, sarcopenic obesity can occur at any BMI, even within the normal weight range [16], making anthropometric assessments especially unreliable tools to classify body composition phenotypes relevant for cancer survival.

## Body mass index

Anthropometric measures such as height and weight are easily gathered and can be collected with reasonable accuracy, making them attractive for large epidemiological studies [14]. BMI is the current standard for the determination of weight status in populations. Individuals may be classified into underweight (<18.5), normal weight (18.5–<25), overweight (25–<30) and obese (≥30) categories; the latter category potentially broken further into Class 1 (30–<35), Class 2 (35–<40), and Class 3 obesity (≥40) [70]. Correlations between BMI and directly-measured percent body fat have been noted to range from 0.58 to 0.75 among individuals without cancer [71], making BMI only moderately associated with adiposity status. Notably, BMI tends to underestimate obesity status when compared to direct measures of adiposity or obesity-related biomarkers [72, 73]. Studies have also shown significant variations in body fat within levels of BMI, which may be particularly true among cancer patients. One study showed that BMI only classified 26% of a cohort of cancer patients as obese while 59% had excess fat mass by direct measure [74]. In this study 31% of those with BMI in the normal range (18.5–<25) had either objectively-measured obesity, low muscle mass, or both, making the normal weight referent category for outcomes analyses a very heterogeneous mix of body composition phenotypes.

A number of systematic reviews have summarized the literature on cancer survival in relation to BMI around the time of diagnosis. A large meta-analysis that involved studies for 15 cancer sites recently considered survival outcomes among those with obesity (BMI ≥ 30) compared to those without obesity. For all cancers combined, authors reported a modestly increased risk of overall mortality (pooled hazard ratio (HR) [95% confidence interval]: 1.14 [1.09, 1.19]) was well as cancer specific death (pooled HR: 1.17 [1.12–1.23]) [1]. Analyses of individual cancers indicated an increased risk of overall mortality for breast, colorectal, and uterine cancer (all HRs around 1.2), while a decreased risk of death among lung, renal cell carcinoma, and melanoma cancer survivors (HRs ranging from 0.74 to 0.86).

Most recently, the Global Cancer Update Program (CUP global) group considered the relationship between a number of anthropometric measures of adiposity and breast cancer outcomes. Elevated BMI was associated with greater all-cause mortality (across 64 studies, pooled relative risk (RR) per 5 kg/m^2^: 1.07 [1.05–1.10]) as well as breast-cancer specific survival (39 studies, RR: 1.10 [1.06–1.14]), recurrence (63 studies, RR: 1.05 [1.03–1.08]), and incidence of second primary cancers (11 studies, RR: 1.14 [1.04–1.26]) [3]. The authors found evidence of a nonlinear “J-shaped” relationship between BMI and survival, with the lowest risk occurring around the threshold for overweight status (BMI 25 vs. 20, RR: 0.95 [0.91–0.99]) with an uptick as BMI increased into the Class 2 obese range (BMI 35 vs. 20, RR: 1.21 [1.12–1.30]). A similar but less pronounced dose-response pattern was also reported for breast cancer-specific survival. In total, these findings were graded as providing “strong” and “probable” evidence for a link between BMI and breast cancer outcomes, while trends regarding recurrence and non-breast cancer related death (including cardiovascular disease) were considered to provide suggestive evidence.

Generally concordant findings have been reported in a recent review of studies focused on colorectal cancer survivors. A similar “J-shaped” relationship between BMI and a number of survival outcomes has been reported among colorectal cancer survivors [2]. Risk of death from any cause was elevated at the extremes of the BMI range; compared to normal weight status those with BMI < 18.5 or ≥35 were at greater risk of death from any cause (summary HR: 1.26 [1.15–1.37] and HR: 1.12 [1.02–1.22], respectively). However, those in the overweight range displayed the lowest risk of death (HR: 0.92 [0.86–0.99]) [2]. Similar patterns were observed for disease free survival and colorectal cancer-specific deaths.

Over the last decade, several other meta-analyses have reported similarly unexpected associations between BMI and cancer-specific survival across different cancer sites. Among individuals with kidney cancer, obesity and overweight status was associated with lower risk of cancer-specific survival compared to those with normal weight status (HR: 0.85 [0.79, 0.93]), but a large increase in risk was noted among underweight individuals (HR: 2.16 [1.15, 4.04]) [75]. A qualitative summary of studies of head and neck cancer survivors showed a similar association, with HRs comparing obesity to normal weight around 0.7 for most reports considered [76]. Notably, these are similar to the magnitude of the pooled HR reported in the sub-analysis of head and neck cancer survivors (HR: 0.59 [0.33–1.05]) in the recent Petrelli meta-analysis [1].

## Circumference measures of central adiposity

Although not as straightforward to measure as height and weight, waist circumference (WC) is a common measure of central adiposity, with higher values tending to indicate a greater deposit of metabolically active visceral fat tissue [77]. Common thresholds for risk stratification by WC are 102 cm for males and 88 cm for females [78]. Despite their greater specificity for capturing regional fat distribution, these measures possess important shortcomings. Although these assessments offer a more refined measure of body composition, they are not always practical, and there can be significant variability across different measurement protocols. Waist circumference also tends to be strongly correlated with overall body size measures such as BMI [79], and so its statistical application as an independent measure of central adiposity requires careful consideration [80]. Furthermore, although WC is driven strongly by visceral fat mass, it cannot distinguish between subcutaneous and visceral adipose tissue around the abdomen. Waist-hip ratio (WHR), a popular alternative to simple WC, may be more problematic, as larger values can be due to greater abdominal adiposity, reduced gluteal muscle mass, or greater subcutaneous fat deposition around the hips and buttocks [14]. These different tissue depots may have important characteristics [81, 82] that become muddled in a single ratio metric.

Studies relating cancer outcomes to anthropometric assessments of central adiposity are much less common in the extant literature. In a recent meta-analysis, Cheng et al. reported moderately strong summary associations between elevated central adiposity and all-cause mortality (pooled HR: 1.30 [1.15–1.46]) and breast cancer-specific death (HR: 1.26 [1.03–1.55]) across 14 studies of breast cancer survivors [83]. For colorectal cancer survival, the authors also noted an increased hazard of all-cause mortality (HR: 1.24 [1.04, 1.47]) and colorectal cancer-specific mortality (HR: 1.27 [1.08, 1.49]) [83]. It is important to note that these pooled estimates included results from studies that used either WC or WHR. As these likely reflect different phenotypes of central adiposity [14], the heterogeneity of the exposure definition obscures the interpretation. However, the previously mentioned meta-analysis of breast cancer outcomes by the CUP global group did conduct separate analyses for WC and WHR and also reported strong evidence for relationships between all-cause mortality and WC (per 10 cm, summary RR: 1.18 [1.07–1.31]) or WHR (per 0.1 unit, RR: 1.30 [1.20–1.40]), with comparable findings for breast cancer-specific deaths [3]. Despite the strength of this evidence, the number of studies summarized for each of these measures in the CUP global study was small, ranging from 3 (WC and breast cancer specific mortality) to 8 (WHR and all-cause mortality). Cheng et al. also report that elevated visceral adiposity is associated with greater overall mortality (summary HR: 1.24 [1.04, 1.47]) and cancer-specific death (HR: 1.27 [1.08, 1.49]) among colorectal cancer patients, but modest to null findings for prostate cancer patients [83].

## Weight change

Weight change is an alternative anthropometric measure of adiposity, as weight gain during adulthood reflects a state of sustained positive energy balance and the accumulation of adipose tissue [80]. Conversely, intentional weight loss among those with excess adiposity has been shown to have beneficial effects on the aforementioned biomarkers of obesity-driven inflammation and insulin resistance [84, 85]. Weight change metrics used by researchers have varied somewhat, and an understanding of the various expressions is required when interpreting findings across the broad literature. Weight change can be derived from expressions of anthropometric variables such as weight (in pounds or kilograms) or BMI, representing absolute or percentage changes over time [86]. Because height scaling equally affects both the numerator and denominator in the calculation of percent change of BMI, it is mathematically equivalent to percent weight change if height is constant over time. However, absolute changes in these variables do not share this property. An important consequence of this difference is that percent weight change over time, as well as absolute BMI change over time, implies larger absolute weight changes for smaller individuals. Taking into account measurement error and normal fluctuations in fluid balance, a threshold of <3% has been proposed for defining weight maintenance [86]. More commonly, an absolute weight change of <5% is used to define weight maintenance in the literature, with losses more than 5% classified as any weight loss, and gains frequently divided into 5– < 10% (moderate gain) and ≥10% (large gain).

Although the implications of weight gain are fairly clear, interpretation of findings related to weight loss in epidemiological studies is challenging, as intentionality is not typically assessed. Intentional weight loss is typically due to the purposeful adoption of dietary and physical activity practices, while unintentional weight loss is thought to reflect disease progression. Notably, weight stability may also not be a reliable measure of ideal body composition, as incident sarcopenia and myosteatosis after diagnosis has been reported among weight stable cancer patients [87]. Therefore, considering weight stable individuals as the reference group in cancer survival studies likely shares the problems noted for the normal weight BMI category [74].

Survival in relation to weight gain after breast cancer diagnosis has been examined extensively in the epidemiological literature. A 2015 meta-analysis of 12 cohort studies of post-diagnosis weight gain and breast cancer survival reported that weight gain of 5% or more from baseline levels was associated with greater risk of mortality from any cause compared to those who maintained weight [88]. However, the more granular analyses presented by the authors showed that this was driven by extreme weight gain (gain of ≥10%, HR: 1.23 [1.09, 1.39]) with a near-null effect for those gaining 5– < 10% of their baseline weight. Notably, the more recent review by the CUP global group concluded that evidence for a link between postdiagnosis BMI or weight changes was inconclusive, and required further study [3].

Among colorectal cancer survivors, a meta-analysis included 3 studies that examined the association between survival endpoints and weight change [89]. The pooled estimates did not suggest an association between any weight gain and overall mortality (pooled HR: 1.03 [0.86, 1.19]) or colorectal cancer-specific survival (HR: 1.02 [0.84, 1.20]) [89]. However, in individual studies, associations were most pronounced between larger weight gain (absolute change of around 5 kg or more) and colorectal cancer-specific survival [90, 91] and overall mortality [90] for pre- to post-diagnosis change, as well as overall mortality for post-diagnosis changes [92]. Although not summarized in that meta-analysis, weight loss in the individual studies was associated with a greater risk of mortality outcomes. Subsequently, in a large health system cohort of colorectal cancer patients, Meyerhardt et al. found a greater risk of cancer-specific death (≥10% loss, HR: 3.20 [2.33, 4.39]) and overall mortality (HR: 3.27 [2.56, 4.18]) among those who lost weight after diagnosis [93]. The association between weight gain and colorectal cancer survival was near-null (≥10% gain HR: 0.93 [0.63, 1.37]), but suggestive for overall mortality (HR: 1.20 [0.91–1.58]). The relationship between weight change and colorectal cancer survival seems to be somewhat more complex than for breast cancer, but indicates a less pronounced convex pattern.

## Imaging based measures of body composition

The advent of methods for body composition assessment based on routine clinical imaging has revolutionized this area of research [94, 95]. These tools offer a number of advantages over anthropometry including not requiring protocols and logistics involved with measurement of anthropometric data, or relying on inaccurate self-reported values. In addition, assembling cohorts of sufficient size is efficient and feasible for such investigations as they use existing images from the medical record [94] and so do not require prospective recruitment. While such studies possess a number of attractive features, their utility in understanding the relationship between cancer outcomes and body composition may be limited to malignancies where imaging in relevant anatomical regions is standard in the course of diagnosis and treatment. These techniques allow for accurate and reliable quantification of the amounts of subcutaneous, visceral, and intra-muscular adipose tissue, as well as skeletal muscle tissue. These are typically measured as cross-sectional areas (cm^2^) from a single image around the L3 vertebra [94]. These quantities are sometimes expressed as index measures by dividing cross-sectional area by squared height to resemble BMI [94]. In addition to quantity, adipose tissue and muscle tissue quality may also be calculated by a measure of tissue radiodensity (in Hounsfield units). Greater adipose tissue radiodensity indicates fat cells with less lipid content and potentially more inflammation, while lower muscle radiodensity reflects more fatty infiltration into a given quantity of muscle tissue, and fewer myocytes.

The review by Cheng et al. recently considered 203 studies across 10 cancer sites for evidence linking imaging-based measures of adipose tissue quantity to cancer progression and survival. With the 128 reports included in the meta-analysis, the authors reported modest to near-null summary HRs across all measures of adipose tissue quantity (area or index measures of total, visceral, and subcutaneous tissue) for most cancer sites [83]. Without regard to statistical significance, a few suggestive associations were also reported: in breast cancer, a greater hazard of overall mortality was observed for subcutaneous fat (pooled HR: 1.36 [0.9, 2.05]), and for progression free survival for visceral fat (HR: 1.20 [0.4, 3.57]) [83]. Interestingly, HR point estimates below the null were observed for visceral or subcutaneous fat for some ovarian and prostate cancer survival outcomes, although the CI crossed the null for these measures. The authors cited study heterogeneity and some methodological limitations as possible factors for the noted inconsistencies.

Adipose tissue quality also seems to be related to mortality, even independent of adipose tissue quantity. In a recent study of colorectal cancer patients, visceral adipose tissue density (VATD) and subcutaneous adipose tissue density (SATD) were positively associated with overall mortality (per 8 HU in VAT: HR: 1.21 [1.11, 1.32]; per 9 HU in SAT, HR: 1.18 [1.11, 1.26]) with similar associations for colorectal cancer mortality [96]. Another report found that that SATD was associated with greater mortality among a cohort of breast cancer patients (high vs. mid SATD values, HR: 1.45 [1.15, 1.81]), while the association with VAT was more modest (high vs. mid VATD, HR: 1.16 [0.90, 1.50]) [97].

A recent review examined the association between muscle and cancer survival, and reported consistent evidence that low muscle quantity was related to poor survival [98]. Unfortunately, differences in study population, methodology, and other factors prevented a formal meta-analysis of these associations. Muscle quality, assessed by skeletal muscle radiodensity is also emerging as an important prognostic factor, with several studies noting inverse associations between muscle density and mortality, sometimes independent of quantity [9, 99–102].

## Discussion

Biological plausibility and volumes of epidemiological evidence suggest a link between body composition and cancer survival, but several issues remain. Reports focused on BMI have been inconsistent and sometimes suggest a contradictory message regarding weight status to what public health officials endorse for general health. The finding that the optimal BMI lies above the upper limit of the normal-weight range in those with chronic disease has been termed the “obesity paradox” or “overweight paradox” which has fueled a robust controversy [17, 103]. However, it should be noted that in this paradox, the nadir of the J-shaped BMI-mortality curve is often shifted to the right rather than suggesting a monotonic decrease in risk with greater BMI (although the latter has been observed). Selection bias has been suggested as the culprit for this paradox [17], but the biasing relationships may have to be unreasonable for this to be the case [104]. Emerging evidence has instead implicated measurement error as the underlying issue, as BMI is a poor metric of the putative aspects of body composition. It has specifically been shown that the observed shifts in the mortality curve may be consistent with opposing relationships between constituent expressions of fat and lean mass [105], and so these observations are not as contrary to general messaging as they might initially appear. In fact, some researchers have proposed this phenomena be re-named the “BMI paradox” to emphasize that the confusion is driven by BMI’s inherent limitations as a relevant measure of adiposity quantity and distribution [106]. Findings for central measures of adiposity, such as WC, and weight gain after diagnosis, which are more precise measures of adiposity, seem to be somewhat more consistent, but the evidence base for these metrics is relatively small.

Imaging-based body composition assessment offers an exciting opportunity to study multiple characteristics of different fat and muscle tissues in clinical populations. To understand the interplay between this myriad of tissue quantity and quality metrics, the analysis of body composition would ideally consider all of these variables together rather than analyzing individual factors in serial fashion. Paradoxical or null relationships between adiposity and survival are often hypothesized to result from potentially protective factors related to the greater muscle mass that tends to occur with higher fat mass [98]. In fact, the recent review of imaging based adiposity and cancer survival pointed out that only 11 of the 128 studies included in the study adjusted for muscle in their individual analyses, with the authors concluding that this potentially contributed to the noted heterogeneity across reports [83]. Simultaneous consideration of all tissues is also suggested by biology, given our understanding of the interrelationships between fat and muscle [49].

Although imaging-based measures of body composition represent an important advancement in the field, some points regarding their interpretation bear mentioning. CT-based body composition measures provide insight into the distribution of fat and muscle in the regions being imaged. Although these metrics correlate strongly with total body volume of these tissues, they do not quantify the exact amount [107]. Another ongoing consideration in imaging-based body composition research is the appropriate expression of tissue quantity that is measured in cross-sectional areas. It has become common, although not standard, for researchers to scale area measures by squared height as in the practice of calculating BMI from weight and height. This results in measures of visceral and adipose tissue index (VATI and SATI, respectively), and skeletal muscle index (SMI). The goal of this transformation is to create measures of body composition quantity that are independent of stature. However, the use of squared height in the calculation of BMI was derived to make the resulting weight-based measure independent of height, which is not without controversy [108]. Interestingly, weight-based measures of body composition, such as total lean mass and total fat mass, do lend themselves to BMI-style normalization by squared height. The resulting metrics, fat mass index (FMI) and lean mass index (LMI) have the appealing property of summing to BMI, thus representing a decomposition into its constituent tissue compartments [109]. It is not clear that applying the same normalization to individual area-based measures achieves the same goal, and other scaling factors may be more appropriate [110]. Ultimately, different uses of area-based and height-adjusted index measures may contribute to the variations noted in the literature on adiposity and survival [83].

Research based on imaging modalities presents challenges to clinical and epidemiological research, some of which have been acknowledged in the radiology literature [111]. However, there are several specifically relevant to opportunistic assessments of body composition in connection with cancer survival. As these metrics are derived from images that are only obtained in the course of the cancer diagnosis, they are unable to be used to characterize the relationship between these body composition features across the entire cancer continuum. Examination of the associations between body composition and incident disease would clarify their relationship to the etiology of cancer subtypes or other factors related to disease severity at diagnosis. As another consequence, if adjustment for body composition before diagnosis is required, researchers are still relegated to the use of anthropometric variables, frequently BMI, which is discordant with the focus on specific fat and muscle characteristics. Furthermore, relatively few patients receive repeated scans, at least to the degree to accurately characterize longitudinal trajectories of body composition. Thus, studies that have utilized repeated body composition assessments have been much smaller than those focused on baseline values [87, 112]. In addition, those with repeated radiological measurements may tend to be less likely to have lower stage disease than those who do not [87], and so results of such analyses may not be generalizable to all cancer patients.

The field of body composition and cancer survival is rapidly evolving with the availability of detailed tissue assessments in clinical populations. Reconciling evidence on body composition from observational studies across a broad array of assessment methodologies is an ongoing challenge when designing meaningful interventions for cancer survivors [95, 113]. While the shift toward more direct measures of body composition for survival research is a clear step forward, researchers and clinicians should to work to identify consistent and meaningful clinical targets that align with general public health guidelines.
